# Extracting for Regulation

**Published:** 1900-06-15

**Authors:** S. J. Rauh


Adjourned.

### EXTRACTING FOR REGULATION.

BY DR. S. J. RAUH.

Read before the Cincinnati Odontological Society.

When, where and how shall we extract, if at all, to insure the best results where regulation is required?

From several cases which have been under my observation within the past few years, some principles are suggested, and the final outcome speaks for itself.

Almost all authorities favor extraction in certain cases, although there are several so extreme in their views as to claim better results without the loss of a single tooth. Theoretically this may be very true, but practically one most important consideration is omitted—the patient. For some inexplicable reason this one consideration forces itself upon us at every operation. When a nervous child is the prospective victim, if good results can be obtained from the extraction of one or more teeth in a crowded arch, decide upon the proper time, and, do it.

The title of this paper had already been selected before an article by Dr. E. A. Bogue, of New York, appeared in the *Cosmos* for December, which was read at the last meeting of the National Association. He takes a very arbitrary stand and says that faulty articulation will result from any extraction, and shows numerous models to prove his point, taken from an experience of many years. That his conclusions, in the main, are correct, our own daily cases will substantiate, such as indiscriminate extraction of first molars, causing the second molars to incline forward, and similar cases.

This, however, need not deter us from extracting a first, or, more rarely, a second bicuspid, to gain the space necessary to allow a cuspid a place in the arch which could only be obtained by expansion of the entire arch.

Reference is made more particularly to that class of cases where the same difficulties would be encountered as are represented by the models shown. The patients were boys of twelve years of age. It is with much difficulty that any operation, no matter how trivial, is completed, and frequently either cement or amalgam is used where gold would have been preferable, proving that anything like a perfect filling would be impossible where the patient was scarcely to be controlled for more than half an hour at a sitting. Gas was administered and the teeth extracted with little or no trouble, the patient not realizing what was to be done.

*Case* 1. Patient, a boy in good health and development, fully up to the average of a person of twelve, which was his age. The original impression was taken in July, 1897 and the teeth extracted in December of the same year.

The teeth which are erupted, it will be noticed, are in good condition, with little decay present. The second molars are not visible either above or below, and only one of the lower bicuspids is seen. The superior cuspids are just beginning to show.

Model number two was obtained just prior to the time of extraction, and the cuspids are plainly seen, with little space for them in the arch. After extraction the patient was instructed to press with the fingers on the cuspids at frequent intervals, so as to mechanically assist them to their proper places. How carefully these instructions were followed it is impossible to state, but the teeth were in proper position some time before the final impressions were taken.

Model number three was obtained in November, 1899, and it will be seen that the results are almost perfect, with little or no trouble to the patient, even the centrals having partly lost that V-shaped tendency which is shown in the original model. The articulation is far better than shown by these models, due to the haste in which they were obtained.

*Case* 2. Upon selecting my models for this case I was much disappointed to find that the original had disappeared. Still, it can be readily seen what the condition was prior to extraction which took place in April, 1897. The condition was very much the same as in the preceding case, except that it existed on only one side of the arch. Model number two, taken in July of the same year, shows the cuspid almost in position.

Model number three, taken November, 1899 an interval of a little over two years, shows the completed case, and the cuspid has completely filled the space caused by the extraction of the bicuspid.

Several cases have been under my observation, similar to those described, and results are the same with but few exceptions, and in these it may be necessary to draw the teeth into position mechanically.

It is rarely that space is required for any of the superior anterior teeth, with the exception of the cuspids, as these will occupy the space intended for the latter before their eruption

The age for extraction would usually be about twelve, just as the cuspids are seen; or, if there be very little space, when the prominence indicating their early eruption is seen or felt.

*Case* 3 Is presented by way of contrast. In this it will be seen that there is more than enough room for the misplaced cuspid, which was very unsightly and caused the lip to protrude. The same directions were given as to pressing on the tooth, with no results; so, after waiting a year with absolutely no movement in the proper direction, regulation was attempted. The patient, a young lady, was very well developed, and it was with some difficulty that the tooth was moved, but after it was once forced into position it was retained easily, and the arch is perfectly normal at present, over a year after the completion of the operation.

Frequently the extraction of one of the four anterior teeth below will remedy a marked irregularity if done at the proper time. The irregularity in this region is mostly confined to the four incisors, and it is seldom that the extraction of either of the first bicuspids would be of any service, that is, without the employment of mechanical means to remedy the defect.

It is not the purpose of this paper to discuss the general rules for extraction as applied to regulating, but merely to show what can be accomplished by judicious extraction at the proper time in special cases.

### DISCUSSION.

DR. CALLAHAN: I have often been in error in extracting for regulating, have had cases where I regretted that I had used the forceps. With experience we come to perceive that often much better results can be achieved by retaining all the teeth in position. It is sometimes absolutely impossible to determine the requirements of a given case from an examination of plaster models. The patient's mouth must be inspected with relation to the irregular positions of the teeth, in order to form an intelligent opinion of the requirements for regulating. If we would take into consideration always the probable future development of both jaws, we would find still less occasion for extraction as a means for attaining the desired end in regulating.

DR. LOCKE: Dr. Rauh has exhibited in one of the models shown, a disadvantage which arises from extracting a first bicuspid in order to make space for a cuspid standing out of line. Now, that procedure (extracting the first bicuspid) is all right, *provided*, the proper appliances are used without delay to complete the correction of the irregularity. But in the case shown, the second bicuspid has been pushed forward probably by the erupting molar, so that the space has become much narrowed. Why did you not go ahead at once to draw the cuspid down into place, Dr. Rauh?

DR. RAUH: I waited to see it come down of its own accord, and so it did to a considerable extent.

DR. LOCKE: Sometimes, if the cuspid is immediately over the first bicuspid, it will drop down all right. But there is an uncertainty about that. I approve of the extraction of the first bicuspid in preference to the second, generally speaking, urging only that the case then be taken in hand without delay. If you extract the second bicuspid, in such a case as this, you may find difficulty in drawing the first bicuspid back into the space, owing to the tendency of the tooth to elongate—to be drawn slightly or considerably from its socket, interference with the natural bite being thus induced.

I am not disposed to agree with Dr. Bogue in the position he takes in his paper, to the effect that extraction is always an error. I think the alternative, widening the arch, is quite often just as objectionable. In many cases where you have been at great pains to widen an arch, in the course of correcting an irregularity, you have given your patient's mouth an unsightly, moon-shaped look. The effect has been to enlarge the mouth, which is often as much of a deformity as the one corrected. Furthermore, the extreme difficulty, not to say impossibility, of preserving a proper articulation, when widening is resorted to, is so great as practically to forbid the operation in many cases.

Dr. Williams: I have sometimes extracted a lower incisor with advantage to a patient annoyed by a crowded condition of those teeth; have gained satisfactory results in the case of a patient as old as twenty-three.

Dr. H. A. Smith: I think it is Dr. Kingsley who says, in his book on regulating, that it is a most difficult problem to decide which tooth to extract. I think there is much experimenting in this matter. To know what tooth to take out, presupposes thorough knowledge of the periods of development and of eruption of teeth, of normal and of abnormal conditions. With reference to the eruption of the deciduous teeth, there is no invariable rule. We think we know something about that, but when we come to look over the tabulated statements of different authors we find ourselves still confronted by uncertainty. Then, absence of teeth is significant, and must be taken into the account. As years go on, the number of these teeth which are missing seems to be on the increase. We see more people to-day than formerly in whom the lateral incisors or the wisdom teeth are missing.

Dr. Wright: At a meeting of the profession in a certain European capital a friend of mine, a foreigner, expressed his unbounded surprise that anyone should openly advocate the extraction of the sixth-year molars. "Why, just think of it," he exclaimed, "what an immense number of teeth which might have been filled, and so have put money into the pockets of us dentists, these fellows are extracting! It is a shame and a disgrace!"

Dr. H. A. Smith: With regard to extraction to correct irregularities, an operation of a novel description, forestalling irregular eruption of the cuspid, has been suggested and actually practiced It is to extract the unerupted first bicuspid, reaching up into the gum after it. This operation is a suggestion of Dr. Guilford's.

Dr. Hunter: I believe that judicious extraction is indicated in more cases than some seem to think. I regard the operation of extracting an unerupted bicuspid as something more readily done on paper than it would ordinarily be found to be in the mouth. As regards expanding the arch, I suspect that not one case in a hundred will admit of both arches being expanded, the articulation being at the same time satisfactorily maintained.

Dr. Rauh: These cases which I have cited to-night were of a description in which, without extraction, nothing whatever could have been done. It was a question of extracting or doing nothing. That is my own view of these cases.

Adjourned.

---

Sulphuric Acid for Alveolar Hemorrhage.—In a case where other well-known methods had failed and the patient was becoming alarmingly weak from loss of blood, sulphuric acid dropped in the socket, after washing the mouth out with warm water, caused the flow to cease within three minutes, and there was no subsequent return. —*R. W. Turner, in Items of Interest.*

